# WHAT’S NEW IN INFECTION ON SURGICAL SITE AND ANTIBIOTICOPROPHYLAXIS IN SURGERY?

**DOI:** 10.1590/0102-672020200004e1558

**Published:** 2021-01-25

**Authors:** Adriano Carneiro da COSTA, Fernando SANTA-CRUZ, Álvaro A. B. FERRAZ

**Affiliations:** 1 Department of Surgery, Federal University of Pernambuco, Recife, PE, Brazil

**Keywords:** Antibiotic prophylaxis, Infection control, Surgical site infection, Postoperative complications, Antibioticoprofilaxia, Controle de infecção, Infecção de sítio cirúrgico, Complicações pós-operatórias

## Abstract

**Introduction:**

Infection of the surgical site is the common complication, with significant rates of morbidity and mortality, representing a considerable economic problem for the health system.

**Objective:**

To carry out a narrative review of the literature on surgical site infection and the principles of antibiotic prophylaxis to update the knowledge of its use in surgery.

**Method:**

Medline, Ovid, Google Scholar, National Library of Medicine (PubMed), Cochrane and SciELO were used for the research. The keywords used were “anti-bacterial agents”; “antibioticoprophylaxis” AND “surgical wound infection”. The inclusion criteria were articles of recent publication, with full texts available and performed in humans.

**Result:**

A total of 29 articles were evaluated and selected according to the eligibility criteria.

**Conclusion:**

Infection of the surgical site is the most common postoperative complication. The key point of its prevention is the combination of several interventions that aim to reduce risk factors, such as: compliance with the new guidelines of the Center for Disease Control and Prevention; the principles of the use of prophylactic antibiotics; factors and risk index of the surgical site; administration time; duration and dosage of antibiotics. These data are available in this article.

## RESUMO

**Introdução:**

A infecção do sítio cirúrgico é a complicação comum, com taxas significativas de morbimortalidade, representando considerável problema econômico para o sistema de saúde.

**Objetivo:**

Realizar revisão narrativa da literatura sobre infecção de sítio cirúrgico e os princípios da antibioticoprofilaxia para atualizar o conhecimento de seu uso em cirurgia.

**Método:**

Utilizou-se para a pesquisa a base de dados Medline, Ovid, Google Scholar, National Library of Medicine (PubMed), Cochrane e SciELO. As palavras-chave usadas foram “anti-bacterial agents”; “antibioticoprophylaxis” AND “surgical wound infection”. Os critérios de inclusão foram artigos de publicação recente, com textos completos disponíveis e realizados em humanos.

**Resultado:**

Um total de 29 artigos foi avaliado e selecionado de acordo com os critérios de elegibilidade.

**Conclusão:**

A infecção do sítio cirúrgico é a complicação pós-operatória mais comum. O ponto-chave da sua prevenção consiste na combinação de várias intervenções que visam reduzir os fatores de risco, tais como: a obediência às novas diretrizes do Centro de Controle e Prevenção de Doenças; aos princípios do uso de antibióticos profiláticos; fatores e índice de risco do local cirúrgico; tempo de administração; duração e dosagem dos antibióticos. Esses dados estão disponíveis neste artigo.

## INTRODUCTION

The surgical site infection (SSI) appears in a wound created by a surgical or post-operative procedure of any cavity, bone, joint, tissue or prosthesis involved. The organisms involved are generally endogenous to the patient and come from the skin or any viscera that has been opened^20^. It is the most common postoperative complication, with significant morbidity and mortality, and represents 17% of healthcare-related infections. Patients with SSI are five times more likely to be readmitted in 30 days and two times more to die, compared to those who do not develop it. In addition, they double hospital stay and costs and, therefore, also represent a considerable economic problem for the health system^2^.

SSI is thus considered if it occurs within 30 days of the operation, or within 90 days when it involves prosthesis implantation, and is classified according to the tissues involved in: 1) superficial incisional, when it involves only skin or subcutaneous tissue at the site of incision; 2) deep incisional, when it covers deep soft tissues (fasciae and muscles); 3) organs and spaces, when it reaches any part of the anatomy other than the incision that was opened or manipulated during the operation^9^.

This study aims to update the theme based on the narrative review of the literature on SSI, and the principles of antibiotic prophylaxis in surgery.

## METHODS

This study constitutes a narrative review on the theme. The database Medline, Ovid, Google Scholar, National Library of Medicine (PubMed), Cochrane and SciELO were used for the research. The keywords used were “surgical site infection”; “antibioticprophylaxis” and “prevention of surgical site infections”. The inclusion criteria were defined: articles of recent publication, with full texts available and performed in humans.

## RESULTS

A total of 29 articles were evaluated and selected according to the eligibility criteria.

### SSI risk factors and index

Antibiotic prophylaxis does not replace any of the other preventive care of SSI and should not be seen in isolation in preventing it, but rather as part of a set of factors based on both the patient and the surgical procedures. Examples of the former may be extremes of age, immunosuppression, diabetes mellitus^12^, perioperative glycemic control, chronic disease^14^, smoking, prolonged hospitalization, MRSA (methicillin-resistant *Staphylococcus aureus*) colonization, coexisting infections in other locations, poor nutritional status or obesity. Factors inherent to the procedures include skin antisepsis, correct surgical technique, adequate hemostasis, maintenance of body temperature, operating time, sterilization of surgical materials and equipment, and positive pressure ventilation in the operating room^13^ ^,^ ^17^ ^,^ ^26^.

The National Academy of Sciences National Research Council has prepared a classification of surgical wounds based on the degree of contamination into four categories: clean, potentially contaminated, contaminated and infected. The data show that these categories have different rates of infection (Table 1).

**TABLE 1:** Classification of surgical wounds and risk of SSI^22^

Class	Contamination potential of the operation	Characteristics	Example	Estimate of occurrence from SSI
I	Clean	No signs of inflammation, no opening of the respiratory tracts, food, genital or urinary	Inguinal herniorrhaphy	<2%
II	Potentially contaminated	Opening of the respiratory tracts, food, genital or urinary with no significant contamination	Cholecystectomy (without overflow bile)	<10%
III	Contaminated	Inflammatory process or opening of the respiratory tracts, food, genital or urinary with significant contamination	Appendectomy, colectomy	20%
IV	Infected	Coarse contamination secondary to pus or drilling	Cholecystectomy by cholecystitis acute with empyema	> 40%

Surveillance systems that track SSI rates have moved away from wound stratifications based on this classification, since they do not account for factors related to the patient and the surgical procedure. The system most used today is the National Nosocomial Infection Surveillance (NNIS) risk index proposed by the Centers for Disease Control and Prevention (CDC) in Atlanta, United States, which is calculated using a scoring system based on three components^2,15, 22^, namely: 1) classification of the patient’s physical state - according to the score of the American Society of Anesthesia (ASA) calculated in the preoperative period and ranging from “one” - for physical normality - to “five”, when the expectation of life is <24 h - scoring 3 for increased risk of SSI; 2) potential for contamination of the surgical wound, which contributed “0” for clean and potentially contaminated operations, and “1” for contaminated and infected operations; 3) duration of the operation, when it exceeds 75% of the estimated time for that type of procedure, the patient receives “1” point through NNIS, that is, the expected duration for appendectomy is 1 h, the colorectal 3 h, and the hepatopancreatic 4 h.

The NNIS surgical infection risk index (composed of the ASA variables, potential for contamination of the surgical wound and duration of the operation) was analyzed according to its categories, and presents the following scores: 0 (three factors absent), 1 (only one present factor), 2 (two factors present), 3 (three factors present), and the higher the score the greater the risk of infection.

### SSI prevention guidelines

The World Health Organization has published updated global guidelines for the prevention of SSI based on 29 items^1^, as well as the guidelines of the Centers for Disease Control and Prevention (CDC) were launched in 1999 and updated in 2017, addressing various measures for the prevention of SSI in the perioperative period^8^. The American College of Surgeons also published the recommendations of the Surgical Infection Society in the same year^5^. Therefore, essentially, surgeons have at least three valid sources of information on best practices to choose from.

The new CDC guidelines have been systematically assessed and classified according to the strength and quality of the available published evidence. The main points are^8^: 1) advise patients to bathe in full body with soap or an antiseptic agent the night before the operation; 2) use alcohol-based intraoperative antiseptic preparation; 3) administer intravenous antimicrobial prophylaxis to obtain adequate serum and tissue concentrations of the drug at the time of opening and closing the surgical incision; 4) do not administer additional antibiotics after skin closure for clean or contaminated procedures, regardless of whether drains have been placed; 5) avoid applying topical ointments, powders or antimicrobial solutions to surgical incisions; 6) maintain perioperative glycemic control in all patients below 200 mg/dl; 7) maintain perioperative normothermia; 8) for patients with normal pulmonary function - who are expected to undergo general anesthesia and endotracheal intubation - increase the fraction of inspired oxygen (FiO2) during the operation and in the immediate postoperative period after extubation; 9) transfusion of blood products should not be avoided in surgical patients as a way to prevent SSI.

### Principles of the use of prophylactic antibiotics

The purpose of antibiotic prophylaxis is to prevent SSI by decreasing the microbial load at the operation site. To avoid it, the tissue concentration must have an effective serum and tissue concentration, that is, above the minimum inhibitory concentration of the antibiotic at the time of the initial skin incision^4^ ^,^ ^11^.

### Indications

Antibiotic prophylaxis should be administered to patients undergoing clean procedures involving prosthesis or implant placement (for example, inguinal hernia with mesh), clean-contaminated and contaminated operations^29^. It should not be used routinely in clean operations, unless they involve the placement of prostheses or implants. Antibiotic prophylaxis should not be used in infected operations, as in this circumstance, effective treatment with antibiotics should be prescribed^17^ ^,^ ^20^ ^,^ ^21^.

### Choice of antibiotic

The antibiotic chosen for prophylaxis should cover the spectrum of most common pathogens that cause SSI. The microorganisms most frequently isolated are those that make up the patient’s microbiota, especially those that make up the skin and the manipulated site. Thus, gram-positive cocci present on the skin, for example *Staphylococcus aureus* and *Staphylococcus* coagulase negative are the most common agents in clean operations, and gram-negative and anaerobic bacteria are present in SSIs after potentially contaminated or contaminated procedures. Generally, low-cost antimicrobial agents with bactericidal properties, narrow spectrum of action, good tissue penetration and covering the pathogens most involved in that particular procedure should be used for antibiotic prophylaxis, in accordance with the recommendations of the hospital infection control committee of that institution^4^ ^,^ ^11^ ^,^ ^17^ ^,^ ^20^.

Cefazolin is the drug of choice for many procedures; it is the most widely studied antimicrobial agent, with proven efficacy in antimicrobial prophylaxis and low cost^11^.

For operations involving the distal intestinal tract, second-generation cephalosporins, such as cefoxitin, are often used for their spectrum of additional anti-anaerobic activity. Patients undergoing splenectomy are at risk of developing infection by encapsulated organisms, and thus should receive vaccination against *Streptococcus pneumoniae*, *Neisseria meningitidis* and *Haemophilus influenzae* type B. In elective operations, this must occur at least two weeks before the operation. In emergency cases, immunizations should ideally be administered two weeks after surgery, when physiological recovery occurs^4^ ^,^ ^11^ ^,^ ^17^ ^,^ ^20^.

### Dosage

The ideal dose should reach and maintain levels of the antibiotic in the blood and tissues that exceed the minimum inhibitory concentration for possible pathogens, from the moment of the incision until the closure of the surgical wound. Therefore, the dose and timing of administration are important. It needs to be individualized based on the patient’s weight; this is particularly important in the context of rising obesity rates worldwide. Currently, the dose of 2 g of cefazolin is recommended for adults, and it should be increased to 3 g in those above 120 kg, to achieve minimum inhibitory concentration values ​​greater than 4 µg/ml. Single dose prophylaxis should always be encouraged, as prolonged time with prophylactic antibiotics increases the risk of adverse effects, and does not provide additional protection. For many of the prophylactic agents used, the first dose does not require adjustment in renal levels; however, subsequent doses, when recommended, may need adjustments^3^ ^,^ ^4^ ^,^ ^11^ ^,^ ^17^ ^,^ ^20^.

### Administration time

Prophylaxis should be started, in almost all circumstances, at least 30-60 min before the skin incision, to ensure that tissue concentrations are reached at the time of the incision. Thus, the antimicrobial agent should be administered as long as it provides serum and tissue concentrations higher than the minimum inhibitory concentration, at the time of the incision and during the surgical procedure. For vancomycin, which requires a long time of administration (1-2 h), the dose should start within 120 min before the incision^11^ ^,^ ^22^ ^,^ ^31^.

### Route of administration

The recommended route of administration is intravenous, because it produces rapid, reliable and predictable serum and tissue concentrations. Some antibiotics reach tissue concentration when administered orally (for example, fluoroquinolones), although there is little data in the literature about its effectiveness in some procedures, such as transrectal prostate biopsy^11^ ^,^ ^17^ ^,^ ^20^.

### Duration

For many procedures, a single dose is adequate, as long as the antibiotic’s half-life covers the period of operation. Additional doses are usually required only for longer operations or when using agents with a short half-life. An additional dose of prophylactic antibiotic is necessary if the operation lasts more than 4 h, and the antibiotic used has a pharmacokinetic profile similar to that of cefazolin. An additional dose should be taken if intraoperative blood loss greater than 1500 ml occurs, as serum concentrations of antibiotics are reduced by blood loss and fluid replacement, leading to levels below the minimum inhibitory concentration of the target bacteria. The duration of antimicrobial prophylaxis should be less than 24 h, as continuity for more than 24 h does not decrease the SSI rates and increase the appearance of antibiotic-resistant bacteria^4^ ^,^ ^11^ ^,^ ^17^ ^,^ ^20^.

Branch-Elliman et al.^10^ analyzed the adverse effects of increasing the length of the antibiotic prophylaxis period by more than 24 h, and demonstrated an increased incidence of adverse events, such as acute kidney injury and infection by *Clostridium difficile*, without increasing protection against infections in addition to contributing to increased antimicrobial resistance.

### Decolonization for methicillin-resistant Staphylococcus aureus

Current evidence and guidelines do not recommend routine screening and eradication for patients colonized by MRSA; however, if in the elective condition he is colonized, he must receive decolonization treatment in the preoperative period. The ideal therapeutic decolonization scheme has not yet been established, but the greatest experience to date has been the use of topical 2% mupirocin, three times a day in intranasal administration, associated with daily baths of 2% chlorhexidine for five days^11,17, 20^.

### Allergy to beta-lactams

Cephalosporins are often the drugs of choice for surgical prophylaxis. Careful medical history should be performed to determine whether the patient has had a true allergic reaction (eg, hives, pruritus, angioedema, bronchospasm and hypotension), as the incidence of true adverse reactions to cephalosporins is rare in patients with allergy to penicillin. In case of allergic to beta-lactams, clindamycin or vancomycin can be used for coverage for gram-positives, associated with aminoglycosides if there is an indication for coverage for gram-negative^11^ ^,^ ^17^ ^,^ ^20^.

## DISCUSSION

### Guidelines for the use of antibiotic prophylaxis

Guidelines were developed jointly by the American Society of Health-System Pharmacists (ASHP), Infectious Diseases Society of America, Surgical Infection Society and the Society for Healthcare Epidemiology of America published in 2013 by ASHP as Therapeutic Guidelines on Antimicrobial Prophylaxis in Surgery. Specific recommendations for the use of antibiotic prophylaxis in surgery are described in Table 2. It should be noted that vancomycin is not recommended as the preferred choice for any procedure. The guideline suggests that it can be included when methicillin-resistant *Staphylococcus aureus* (MRSA) is detected in an institution or in colonized patients and should be considered for patients at high risk for MRSA colonization. The guideline panel highlights that vancomycin is less effective than cefazolin in preventing postoperative infections caused by *Staphylococcus aureus* methicillin sensitive; thus, vancomycin is used in combination with cefazolin in some institutions. When used, a single dose (15 mg/kg) is generally acceptable, due to its long half-life^4^ ^,^ ^11^.

**TABLE 2:** Recommendations for antibiotic prophylaxis for surgical procedures^4^ ^,^ ^11^

Operation type	Antibiotic recommended	Usual dose in adults (IV)	Dose additional intraoperative	Duration
Herniorraphy with mesh	Cefazolin	<120 kg=2 g /≥120 kg=3 g	4 h	Single dose
Surgery gastroduodenal	Cefazolin	<120 kg=2 g / ≥120 kg=3 g	4 h	Single dose
Surgery biliopancreatic	Cefazolin	<120 kg=2 g / ≥120 kg=3 g	4 h	Single dose
	Cefazolin	2 g	2 h	
	Ampicillin-sulbactam	3 g	2 h	
Appendectomy and colorectal surgery	Cefazolin	2 g	2 h	Duration = 24 h
	Cefazolin	<120 kg=2 g / ≥120 kg=3 g	4 h	
	+			
	Metronidazole	500 mg	NA	
	Ampicillin-sulbactam	3 g	2 h	

IV=intravenous; NA=does not apply; for patients allergic to penicillins and cephalosporins, clindamycin (900 mg) or vancomycin (15 mg/kg IV; do not exceed 2 g) is recommended, with gentamicin (5 mg/kg, IV); or aztreonam (2 g, IV).

### Bolus vs. antibiotic prophylaxis continuous infusion

Recent studies have shown better results for the use of continuous prophylactic antibiotic infusion when compared to intermittent bolus infusion. Naiket al.^25^ in a randomized study, evaluated the infusion in intermittent bolus of cefazolin (2 g every 4 h), compared with the continuous (500 mg per hour), and demonstrated that continuous intraoperative infusions of cefazolin provide better plasma concentrations, even with lower doses of infusion.

Skhirtladze-Dworschaket al.^28^ compared antibiotic prophylaxis with cefuroxime in intermittent bolus and continuous infusion assessing their serum and subcutaneous tissue concentrations; observed higher concentrations of cefuroxime and for a longer period of time in the plasma and subcutaneous tissue, when cefuroxime was administered continuously, and concluded that its concentration measurements were higher in patients who received the antibiotic in continuous infusion.

Ferraz et al.^18^ studied the continuous infusion of cefazolin vs. ampicillin/sulbactam and ertapenem in bariatric patients, and evaluated their effects on the incidence of surgical site infection. The infection rate was analyzed, as well as its association with age, gender, preoperative weight, body mass index and comorbidities, noting that the SSI rates were 4.16% in the group prophylactically treated with ampicillin/sulbactam, 1.98% for ertapenem and 1.55% for continuous cefazolin. They concluded that prophylactic use of cefazolin in continuous infusion shows very promising results.

Shoulderset al.^27^ studied the impact on SSI incidence of antibiotic prophylaxis vs. continuous infusion of cefazolin vs. intermittent bolus infusion. A total of 516 adult patients received cefazolin intraoperatively, of which 284 in an intermittent bolus and 232 in continuous infusion. Superficial SSIs were significantly reduced in patients who received antibiotic prophylaxis in the form of continuous infusion (2.8% in intermittent vs. 0.4% in continuous, p=0.039).

### New technologies and research in the prevention of SSI

Interest in new technologies and scientific research to help prevent infections is growing. Although there is substantial promise to improve results with new technologies, the decrease in infection rates in the clinical setting has not been well established. However, they are an attractive complement to infection prevention programs.

Extensive research using various methodologies has been carried out with operating room disinfection systems using machines emitting ultraviolet light and hydrogen peroxide; however, the real clinical benefit remains questionable, in addition to the high cost, and especially when traditional human cleaning practices can be optimized^16^. Some clinical studies have shown that devices with ultraviolet light and hydrogen peroxide systems, when used to disinfect operating rooms, can reduce colonization or infections in inpatients^30^. Fu et al.^19^ reported complete eradication of methicillin-resistant *Staphylococcus aureus* (MRSA), *Acinetobacter* and *Clostridium difficile* using a vaporized hydrogen peroxide device.

Another point that must be considered is the unmet medical need for new measures to prevent SSI by *Staphylococcus aureus*. Vaccination against infection by it is still in veterinary use, and has not been successful so far in humans, despite several attempts^6^ ^,^ ^24^.

A very promising multicentre European study (SALT Study) is being conducted to determine the general and specific risk of SSI procedures for *Staphylococcus aureus*, but its results have not yet been published. Advances in the understanding of the pathophysiology of SSI by this microorganism and in the identification of patients at greatest risk, may guide the design of clinical trials for research with effective vaccines against it^23^.

There is limited data in the literature on topical administration of antibiotics to supplement the intravenous regimen and has not demonstrated safety or efficacy. Bennett-Guerrero et al^7^ studying the prophylactic use of topical antibiotics through the implantation of collagen sponges with gentamicin above the fascia at the time of surgical closure in patients undergoing colectomy showed that the incidence of infection in the surgical site was higher in the group that used the sponge.

The safety and efficacy of topical antimicrobials have not been clearly established and, therefore, the routine use of this route cannot be recommended^11^.

## CONCLUSION

Infection of the surgical site is the most common postoperative complication. The key point of its prevention is the combination of several interventions that aim to reduce risk factors, such as: compliance with the new guidelines of the Center for Disease Control and Prevention, the principles of the use of prophylactic antibiotics, factors and index of risk of SSI, time of administration, duration and dosage of antibiotics, data that are available in this article.
